# Integrated Analysis of m6A Methylome in Cisplatin-Induced Acute Kidney Injury and Berberine Alleviation in Mouse

**DOI:** 10.3389/fgene.2020.584460

**Published:** 2020-11-20

**Authors:** Jianxiao Shen, Wanpeng Wang, Xinghua Shao, Jingkui Wu, Shu Li, Xiajing Che, Zhaohui Ni

**Affiliations:** ^1^Department of Nephrology, Renji Hospital, Shanghai Jiao Tong University School of Medicine, Shanghai, China; ^2^Department of Nephrology, Lianshui People’s Hospital, Lianshui, China

**Keywords:** M6A, cisplatin induced nephrotoxicity, berberine, FGA, SLC12A1, Havcr1

## Abstract

**Background:**

N6-methyladenosine (m6A) is the most abundant modification known in mRNAs. It participates in a variety of physiological and pathological processes, such as metabolism, inflammation, and apoptosis.

**Aims:**

To explore the mechanism of m6A in cisplatin-induced acute kidney injury (AKI) and berberine alleviation in mouse.

**Methods:**

This study investigated the N6-methyladenosine (m6A) methylome of kidneys from three mouse groups: C57 mice (controls), those with CI-AKI (injury group, IG), and those pretreated with berberine (treatment group, TG). Methylated RNA Immunoprecipitation Next Generation Sequencing (MeRIP-seq) and RNA-seq were performed to identify the differences between the injury group and the control group (IvC) and between the treatment group and the injury group (TvI). Western blotting was performed to identify the protein levels of candidate genes.

**Results:**

In IvC, differentially methylated genes (DMGs) were enriched in metabolic processes and cell death. In TvI, DMGs were enriched in tissue development. Several genes involved in important pathways related to CI-AKI showed opposite methylation and expression trends in the IvC and TvI comparisons.

**Conclusion:**

m6A plays an important role in cisplatin induced AKI and berberine may alleviate this process.

## Introduction

Cisplatin is an anticancer drug widely used for the treatment of various solid tumors, but it is also known for its nephrotoxicity. It exerts its function by interacting with and disrupting DNA and mitochondrial function. During drug metabolism, cisplatin accumulates in renal tubular cells, causing cell death and resulting in acute kidney injury (AKI) (Lebwohl and Canetta, 1998; Cummings and Schnellmann, 2002; Ozkok and Edelstein, 2014; George et al., 2018; Holditch et al., 2019; Yimit et al., 2019). Recent studies have revealed that apoptosis, necrosis, inflammation, and other mechanisms play significant roles in cisplatin-induced AKI (Sahu et al., 2015; Zuk and Bonventre, 2016; Humanes et al., 2017; Long et al., 2017).

Berberine is the principal component of many popular medical plants, such as *Coptis chinensis*, *Rhizoma coptidis*, *Hydrastis canadensis*, *Berberis aquifolium*, and *Berberis vulgaris* (Wang et al., 2017; Duan et al., 2018; Fan et al., 2019). As a promising drug, various pharmacological activities of berberine have been reported, including antimicrobial, antiemetic, antipyretic, anti-pruritic, antioxidant, anti-inflammatory, hypotensive, anti-arrhythmic, and sedative activities (Shamsa et al., 1999; Chen et al., 2012; Caliceti et al., 2016). Several reports have mentioned that berberine shows nephroprotective effects against cisplatin-induced kidney damage (Domitrović et al., 2013; Teng et al., 2015; Ojha et al., 2016; Ahmad et al., 2019). However, the mechanism of alleviating CI-AKI is still unclear.

m6A has been implicated in all aspects of posttranscriptional RNA metabolism, such as regulating reversible modifications, alternative splicing, stability, and translation. It prefers to modify sequences identified as RRACH, where R is adenine or guanine, A is the m6A site, and H is adenine, cytosine, or uracil. In addition, m6A modifications exhibit enrichment in the 3’ UTR near mRNA stop codons and within long internal exons. The writing of m6A is accomplished via a complex composed of methyltransferase-like 3 (METTL3), methyltransferase-like 14 (METTL14), and Wilms tumor 1-associated protein (Ping et al., 2014; Wang et al., 2014; Spitale et al., 2015). Two m6A demethylases, fat mass and obesity-associated protein (FTO) and AlkB homolog 5, have been discovered as “erasers” (Jia et al., 2011). While proteins containing the YT521-B homology (YTH) domain, such as YTHDC1, YTHDF1, YTHDF2, and YTHDF3, directly bind m6A sites and act as readers of the m6A signal (Zheng et al., 2013).

Several studies have focused on the correlation between m6A methylation and kidney injury. Wang J. et al. (2019) reported that METTL3 overexpression plays a protective role against colistin-induced oxidative stress and apoptosis in renal tubular epithelial cells in mice. Alteration of m6A regulators was associated with pathologic stage in patients with clear cell renal cell carcinoma (Zhou J. C. et al., 2019). Another study reported that cisplatin treatment reduced FTO expression and increased m6A levels *in vivo* and *in vitro*. They also found that inhibiting FTO by meclofenamic acid aggravated renal damage and increased apoptosis in cisplatin-treated kidneys (Zhou P. et al., 2019). METTL14 is elevated in people with AKI (Xu et al., 2020). However, few studies have investigated the m6A methylome in cisplatin-induced AKI and the potential mechanism of berberine alleviation.

In this study, we found that berberine significantly alleviated cisplatin-induced AKI in a reliable mouse model. To further investigate the role of m6A in this process, MeRIP-seq was used to establish the first known transcriptome-wide m6A methylome profiles of kidneys from normal, CI-AKI, and berberine-pretreated mice. RNA-seq was performed to detect differentially expressed genes (DEGs) among the groups. Based on our results, we speculate that berberine may alleviate CI-AKI by regulating m6A methylation.

## Materials and Methods

### Animals and Tissue Collection

Male C57BL/6 mice (aged 8 weeks) were randomly assigned to the control group, injury group (IG), and treatment group (TG), with four mice per group. All mice were housed under a 12 h light/dark schedule with free access to food and water. Control and IG mice were subjected to daily intraperitoneal (i.p) injections with vehicle (normal saline), while TG mice were injected daily with berberine (Sigma, St. Louis, MO, United States, 20 mg/kg) (Ruan et al., 2017). After 14 days of drug treatment, the IG and TG were injected intravenously with cisplatin (20 mg/kg), while controls were injected intravenously with a vehicle. After cisplatin injection, berberine pretreatment was stopped and all mice were housed as usual. All mice in the three groups were sacrificed at 72 h postinjection by cervical dislocation after CO_2_-induced narcosis (Zhang et al., 2016; Dutta et al., 2017). Immediately afterward, the kidneys were collected.

### Serum Levels of Creatinine and Blood Levels of Urea Nitrogen

Before the mice were sacrificed, blood was drawn from their tail veins. Serum samples were collected. Serum levels of creatinine (Scr) and urea nitrogen (BUN) were analyzed using a standard spectrophotometric assay (Roche Diagnostics GmbH, Mannheim, Germany).

### Histopathology Analyses

Renal tissue harvested from animals was washed with 0.9% saline, fixed in 10% neutral buffered formalin, and then embedded in 10% paraffin. Sections (5 μm thick) were stained with hematoxylin eosin for further microscopic analyses. A tubular injury score was calculated (Leemans et al., 2005). The percentage of damaged tubules in the corticomedullary junction was estimated by a nephropathologist using a 5-point scale according to the following criteria: tubular dilation, cast deposition, brush border loss, and necrosis in eight randomly chosen, non-overlapping fields (×400 magnification). Lesions were graded on a scale from 0 to 5: 0 = normal; 1 = mild, involvement of less than 10% of the cortex; 2 = moderate, involvement of 10–25% of the cortex; 3 = severe, involvement of 25–50% of the cortex; 4 = very severe, involvement of 50–75% of cortex; 5 = extensive damage, involvement of more than 75% of the cortex.

### RNA MeRIP-seq and Data Analyses

In accordance with the manufacturer’s instructions, TRIzol reagent (Invitrogen Corporation, CA, United States) was used to extract total RNA from kidney tissue. Ribosomal RNA was removed from total RNA with a Ribo-Zero rRNA Removal Kit (Illumina, Inc., CA, United States). Then, fragmentation buffer (Illumina, Inc.) was used to split the RNA into fragments of approximately 100 nucleotides in length. MeRIP-Seq was performed by Cloudseq Biotech Inc. (Shanghai, China). In brief, total RNA was extracted from kidney tissue using TRIzol Reagent (Life Technologies CA, United States). RNA was tested for quality via NanoDrop (Thermo Fisher Scientific, MA, United States). RNA integrity was assessed using a denaturing agarose gel. mRNA was isolated from total RNA using a Seq-Star^TM^ poly(A) mRNA Isolation Kit (Arraystar, MD, United States). The GenSeq^TM^ m6A RNA IP Kit (GenSeq Inc., China) was used to perform m6A RNA immunoprecipitation. A NEBNext^®^ Ultra II Directional RNA Library Prep Kit (New England Biolabs, Inc., MA, United States) was used to construct both the input samples without immunoprecipitation and the m6A IP samples. All samples were subjected to 150 bp paired-end sequencing on an Illumina HiSeq instrument (Illumina, Inc.).

Paired-end reads were quality controlled by Q30. Trimming of the 3’ adaptor and low-quality read removal were performed using Cutadapt software (v1.9.3) (Kechin et al., 2017). Hisat2 software (v2.0.4) (Kim et al., 2015) was used to align clean reads of all libraries to the reference genome (mm10). Then, methylated sites on RNAs (peaks) were identified using Model-based Analysis of ChIP-Seq (MACS) software (Zhang et al., 2008; Li et al., 2017; Huang et al., 2019). Identified m6A peaks were subjected to motif enrichment analyses using Hypergeometric Optimization of Motif EnRichment software (Heinz et al., 2010), and metagene m6A distribution was characterized using R package MetaPlotR (Olarerin-George and Jaffrey, 2017). Differentially methylated sites with a fold change cutoff of ≥ 2 and *P* < 0.05 were identified using the diffReps differential analysis package (Shen et al., 2013; Wang Y. et al., 2019; Wang et al., 2020). The peaks identified by MACS and diffReps overlapping with exons of mRNA were selected for further analyses. Gene ontology (GO) and pathway enrichment analyses were performed on the differentially methylated proteins for using the GO^1^ and Kyoto Encyclopedia of Genes and Genomes (KEGG)^2^ databases.

### RNA-Seq and Data Analyses

Total RNA was extracted from kidney samples using TRIzol reagent (Life Technologies) according to the manufacturer’s protocol. Denaturing agarose gel electrophoresis was used to evaluate the integrity of total RNA. A Seq-Star TM poly(A) mRNA Isolation Kit (Arraystar, MD, United States) was utilized to purify mRNA from total RNA after measuring the quantity and quality on a NanoDrop ND-2,000. Then, a BGISEQ-500 platform was used to subject fragmented mRNA to 50 bp single-end sequencing. Adapter and low-quality reads were trimmed using SOAPnuke (Chen et al., 2018), and those trimmed reads were aligned to the reference genome using bowtie2 (Langmead and Salzberg, 2012). Finally, cuffdiff was use to analyze differential expressed genes (DEGs) (Trapnell et al., 2012).

### Western Blotting

Mouse tissues were lysed using a protein lysis buffer containing 20 mM Tris (pH 7.4), 150 mM NaCl, 1 mM EDTA, 1 mM EGTA, 1% Triton X-100, 25 mM sodium pyrophosphate, and 2 mM sodium orthovanadate aprotinin. All denatured proteins were separated on an SDS-PAGE gel and then transferred to polyvinylidene difluoride membranes (Roche, Netley, NJ, United States). The membranes were blocked with 5% skimmed milk in Tris-buffered saline and then were incubated with 1:500 dilutions of primary antibodies as follows: anti-FGA (Abcam, Cambridge, MA, United States), anti-Slc12a1 (Abcam, Cambridge, MA, United States), and anti-Havcr1 (Abcam, Cambridge, MA, United States). Then, the samples were incubated with a horseradish peroxidase-conjugated anti-rabbit secondary antibody (Jackson ImmunoResearch, PA, United States). The bands were visualized using an ECL Western Blotting Kit (Biovision, Milpitas, CA, United States) and were quantified by Quantity One software (Bio-Rad, Hercules, CA, United States).

### Statistical Analyses

Data are expressed as mean ± standard deviation (SD). Student’s *t*-test was used to compare two groups. ANOVA with Tukey’s post-test was used to assess the statistical significance between-group means for comparisons between multiple groups. The differences were considered statistically significant at *P* < 0.05.

## Results

### Establishment of a Reliable CI-AKI Mouse Model

**FIGURE 1:**
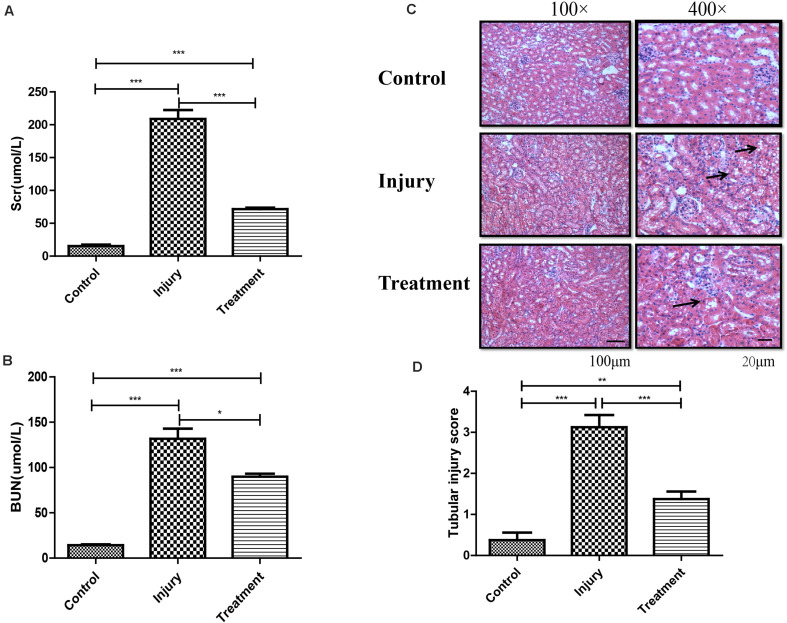
Establishment of cisplatin-induced acute kidney injury model in C57 mouse. **(A)** Analysis of Scr level in mice following different treatments. Error bars represent the standard deviation. ***p < 0.001, Student’s *t*-test. **(B)** Analysis of BUN level in mice following different treatments. Error bars represent the standard deviation. *p < 0.05, ***p < 0.001, Student’s *t*-test. **(C)** Represents the image of hematoxylin and eosin staining in kidney (black arrows indicating the injury). **(D)** Score for characteristic histologic signs of renal injury. **p < 0.01, ***p < 0.001, Student’s *t*-test.

We found that 20 mg/kg cisplatin injection resulted in an approximately 18–20-fold increase in Scr and BUN relative to the control group, but significantly lower levels in TG than in IG (Figures 1A,B). Hematoxylin–eosin staining indicated that cisplatin induced remarkable renal structure damage in IG tissue, including extensive tubular vacuolization, tubular epithelial cell exfoliation, and thickening of glomerular basement membrane (*P* < 0.001), while these changes were notably alleviated in TG mice (*P* < 0.001) (Figures 1C,D). This indicated successful and reliable establishment of a CI-AKI mouse model and relief of the injury through berberine pretreatment.

### General Features of m6A Methylation

**FIGURE 2:**
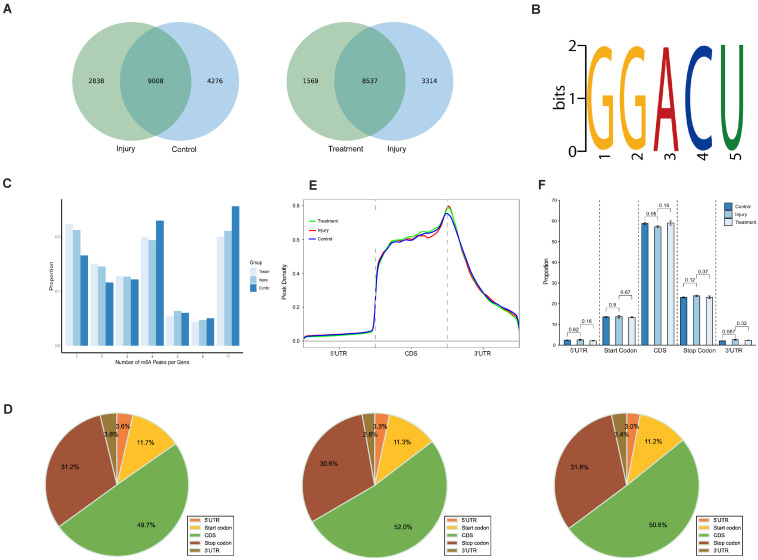
Overview of N6-methyladenosine methylation within mRNAs in the control, injury, and treatment groups. **(A)** Venn diagram showing the overlap of m6A peaks within mRNAs between IvC (left) and TvI (right). **(B)** The top motif enriched across m6A peaks identified from three groups. **(C)** Proportion of genes harboring different numbers of m6A peaks in three groups. The majority of genes harboring more than one m6A peak. **(D)** Pie charts showing the percentage of m6A peaks in five segments of transcripts. m6A peaks were most enriched in the coding sequence segment. **(E)** Distributions of fold enrichment of m6A peaks in five segments. The mean fold enrichment in the stop codon segments was the highest in injury group, while that value in CDS was the largest in treatment group. **(F)** Analysis of m6A peaks in different groups. Error bars represent the standard deviation. Student’s *t*-test.

MeRIP-seq analyses of mRNA derived from kidneys revealed 13,284 m6A peaks within 7,942 coding gene transcripts in controls; the values were 11,846 within 7,422 mRNAs in IG and 10,106 within 6,481 mRNAs in TG. Overall, 7,337 peaks overlapped among the three groups (Supplementary Figure 1). Pairwise comparisons showed that 9,008 peaks (more than 55.8% of all peaks) overlapped in IG versus control (IvC) comparisons and 8,537 peaks (more than 63.6% of all peaks) overlapped in TG versus IG (TvI) comparisons (Figure 2A). Approximately 40% of all peaks were non-overlapping, suggesting differences among those groups.

To determine whether the m6A peaks contained the RRACH conserved sequence motif, we detected all samples in three groups. One thousand peaks with the highest scores (−10^∗^log10, *p*-value) were analyzed using DREME software. The results showed that the same sequence motif (RRACH) was necessary for m6A methylation in kidney mRNAs in each group (Figure 2B), consistent with other studies (Dominissini et al., 2012; Meyer et al., 2012; Luo et al., 2019).

Further analyses demonstrated that 16.59, 21.21, and 22.29% of the genes with m6A-methylation sites in controls, IG, and TG contained one m6A peak (Figure 2C). More than 75% of the genes contained two or more peaks (Figure 2C). This result is different from other reports on mouse liver (Luo et al., 2019) and brain (Dominissini et al., 2012), suggesting that the kidney is unique.

To understand the preferential locations of m6A peaks, all peaks were categorized into five transcript segments: the 5’ UTR, start codon segment, coding sequence (CDS), stop codon segment, and 3’ UTR. We found that m6A was mostly enriched in the CDS, and there was some enrichment near stop codons (Figure 2D). These results are also similar previous studies (Dominissini et al., 2012; Meyer et al., 2012).

Furthermore, m6A peaks in all groups had the highest density in stop codons (Figure 2E). Comparisons between different groups showed no significant difference in the volume of m6A peaks, which indicates that although many variants in m6A methylation were detected in different segments, the total proportion of m6A peaks did not significantly change (Figure 2F).

### Distribution of Differentially Methylated m6A Sites

In IvC, we found 2,981 differentially methylated m6A sites (DMMSs) within 2,227 genes, of which 48.17% (1,436/2,981) exhibited significant increases in methylation. Table 1 displays the top 20 differentially methylated m6A sites.

**TABLE 1:** The top 20 differently methylated m6A peaks in IvC.

Gene name	Gene ID	Fold change	Regulation	Chromosome	Peak start	Peak end	Peak length	*p*-value
BC061237	385138	1489	Up	chr14	44504106	44504291	185	1.48521E-12
BC061237	385138	816.8	Up	chr14	44500121	44500197	76	3.23828E-13
Krt20	66809	498.7531381	Up	chr11	99430752	99430920	168	9.96013E-10
Krt20	66809	498.0274348	Up	chr11	99429021	99429078	57	9.08594E-10
Krt20	66809	483.5165816	Up	chr11	99432181	99432343	162	2.59398E-09
1700001F09Rik	71826	383.8	Up	chr14	43346701	43346790	89	5.98092E-15
Ccdc85b	240514	328.5877193	Up	chr19	5454141	5454580	439	3.35868E-11
Gm3543	100041849	318	Up	chr14	41982201	41982290	89	1.38739E-12
Serpina3n	20716	316.3	Up	chr12	104414261	104414329	68	7.77029E-14
Gm3543	100041849	254	Up	chr14	41982133	41982180	47	4.03611E-10
Alms1	236266	503.6	Down	chr6	85694833	85694951	118	4.2803E-10
Tas2r119	57254	385.6	Down	chr15	32177288	32177620	332	9.86777E-14
Ctnna2	12386	220.8	Down	chr6	77600041	77600380	339	1.36972E-13
Afm	280662	195.1	Down	chr5	90518931	90519060	129	1.23171E-10
Slc5a4a	64452	126.7	Down	chr10	76163688	76163769	81	7.19208E-10
Nat1	17960	123.5	Down	chr8	67490861	67491460	599	2.54765E-10
Pzp	11287	111.1290323	Down	chr6	128526621	128526720	99	8.47489E-15
Dpf3	70127	99.2	Down	chr12	83215461	83215800	339	3.4897E-12
Dgkg	110197	95.3	Down	chr16	22479365	22479426	61	1.61986E-10
Dpf3	70127	94.3	Down	chr12	83214541	83214840	299	1.03615E-10

**FIGURE 3:**
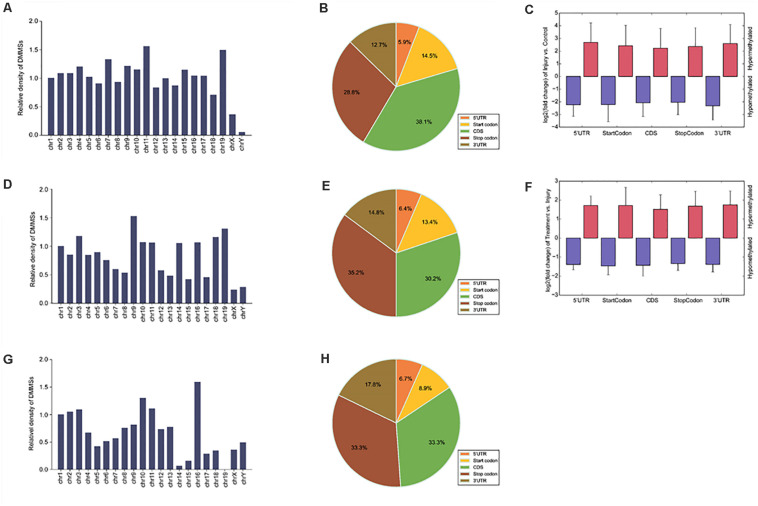
Distribution of differentially methylated N6-methyladenosine sites. **(A)** Relative occupancy of differentially methylated m6A sites in each chromosome normalized by length in IvC. **(B)** Pie chart showing the percentage of DMM peaks in five non-overlapping segments in IvC. **(C)** Statistics of fold change of DMM peaks in five segments. The histogram shows the mean of the fold change in IvC. Error bars represent the standard error of the mean. **(D)** Relative occupancy of differentially methylated m6A sites in each chromosome normalized by length in TvI. **(E)** Pie chart showing the percentage of DMM peaks in five non-overlapping segments in TvI. **(F)** Statistics of fold change of DMM peaks in five segments. The histogram shows the mean of the fold change in TvI. Error bars represent the standard error of the mean. **(G)** Relative occupancy of differentially methylated m6A sites in each chromosome normalized by length. **(H)** Pie chart showing the percentage of DMM peaks in five non-overlapping segments.

To understand the DMMS distribution profiles in those two groups, we mapped them to chromosomes. Chromosomes 4, 2, 7, and 11 were the top four chromosomes, harboring more than 200 DMMSs (Supplementary Figure 2A). However, when the number of DMMSs was normalized by the length of chromosomes, the top four with the highest relative densities were 11, 19, 7, and 4 (Figure 3A). Meanwhile, most DMMSs were within a CDS (Figure 3B). Further analyses showed that at sites with increased methylation, DMMSs within the 5’ UTR had the highest fold change, while among the sites with decreased methylation, DMMSs within the 3’ UTR had the highest fold change (Figure 3C), demonstrating the location preferences of methylation and demethylation within the genome.

In TvI comparisons, 526 DMMSs were identified within 420 genes, of which 66.73% (351/526) were sites with increased methylation. The top 20 m6A sites with the most increased and decreased methylation are shown in Table 2.

**TABLE 2:** The top 20 differently methylated m6A peaks in TvI.

Gene name	Gene ID	Fold change	Regulation	Chromosome	Peak start	Peak end	Peak length	*p*-value
Syt15	319508	63.6	Up	chr14	34228038	34228320	282	1.59789E-06
Celf5	319586	48.7	Up	chr10	81469443	81469523	80	1.58151E-06
Cav3	12391	46.9	Up	chr6	112459821	112459925	104	3.0379E-06
Npffr2	104443	44.1	Up	chr5	89582741	89583200	459	5.67547E-06
Kcnf1	382571	19.08571429	Up	chr12	17174961	17175260	299	3.61832E-06
Amy2a4	100043684	18.27027027	Up	chr3	113279836	113279860	24	1.19296E-06
Metap2	56307	18.03125	Up	chr10	93858488	93858660	172	2.64599E-06
Tbc1d10c	108995	17.81081081	Up	chr19	4184361	4184740	379	4.61772E-06
Cx3cr1	13051	16.29090909	Up	chr9	120051221	120051460	239	1.12074E-09
Bhlhe22	59058	16.16216216	Up	chr3	18055801	18056180	379	1.39035E-06
Fgg	99571	12.77879342	Down	chr3	83008647	83008741	94	2.1945E-09
Fgg	99571	11.67778106	Down	chr3	83010063	83010180	117	1.6791E-09
Dvl3	13544	7.87254902	Down	chr16	20522461	20522660	199	8.52622E-06
Fgb	110135	7.239726027	Down	chr3	83049701	83049803	102	2.426E-08
Slc2a5	56485	6.895348837	Down	chr4	150143641	150143960	319	9.64308E-09
Gm21994	102637277	6.525504152	Down	chr2	150254517	150255160	643	3.39444E-07
Fga	14161	5.909136445	Down	chr3	83031021	83031620	599	1.55539E-08
Fga	14161	5.251931475	Down	chr3	83026152	83026260	108	8.18498E-12
Mpped1	223726	5.03943662	Down	chr15	83856361	83856840	479	6.39334E-11
Calcb	116903	4.906439854	Down	chr7	114721974	114722161	187	6.45098E-12

Chromosomes 3, 1, 9, and 4 were the top four chromosomes harboring the most DMMSs (Supplementary Figure 2B). The top four with the highest relative densities were 9, 19, 18, and 3 (Figure 3D). Most DMMSs were within a CDS (Figure 3E). DMMSs within the 3’ UTR had the highest fold change among sites with increased methylation, while those near the start codon had the highest fold change among sites with decreased methylation (Figure 3F).

We further analyzed DMMSs with contrary methylation trends in IvC and TvI. In total, 94 DMMSs showed opposite trends. Of these, 42.55% (40/94) exhibited significantly increased methylation in IvC but decreased methylation in TvI.

Chromosomes 1, 2, 11, and 10 were the top four chromosomes harboring the most DMMSs (Supplementary Figure 3). The top four with the highest relative densities were 16, 10, 11, and 3 (Figure 3G). Most DMMSs were within a CDS (Figure 3H).

### Differentially Methylated RNAs Are Involved in Important Biological Pathways

To explore the role of m6A in different groups, GO enrichment analyses and KEGG pathway analyses were used to analyze all protein coding genes containing DMMSs.

**FIGURE 4:**
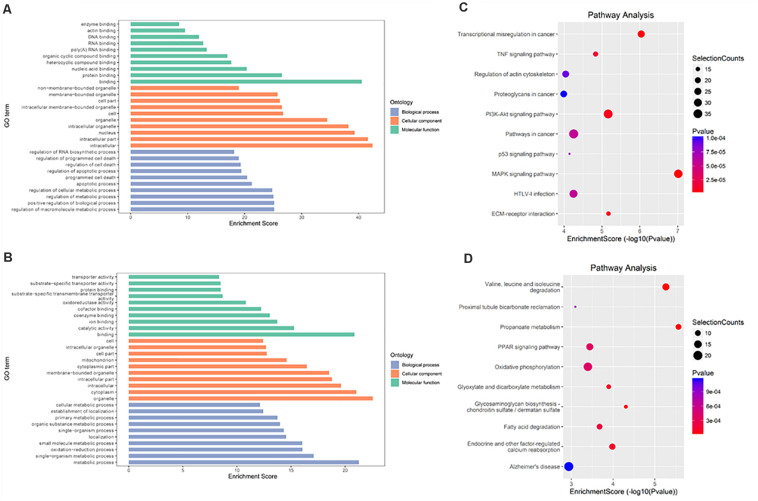
Gene ontology and Kyoto Encyclopedia of Genes and Genomes analyses of coding genes containing DMMSs in IvC. **(A)** Major gene ontology terms were enriched for the genes containing up-regulated m6A sites in IvC. **(B)** Major gene ontology terms were enriched for the genes containing down-regulated m6A sites in IvC. **(C)** Major enriched pathways for the genes containing up-regulated m6A sites in IvC. **(D)** Major enriched pathways for the genes containing down-regulated m6A sites in IvC.

In IvC (Figure 4), for the BP category, genes with increased methylation of m6A sites were significantly (*p* < 0.05) enriched in the regulation of metabolic processes and cell death processes, such as macromolecule metabolic, cellular metabolic, and apoptotic processes (Figure 4A); genes with decreased methylation were highly enriched in metabolic processes, oxidation–reduction processes, transport, transmembrane transport, and others (Figure 4B). Regarding pathways, the former were significantly involved in apoptosis-associated pathways (e.g., TNF, MAPK, P53 signaling pathways) while the latter were involved in propanoate metabolism, oxidative phosphorylation, and others (Figures 4C,D). For the CC category, genes containing DMMSs were mainly enriched in the intracellular organelles, cytoplasm, and nucleus intracellular membrane-bound organelles. For the MF category, upregulation of m6A was notably enriched in genes involved in enzyme binding, actin binding, DNA binding, and RNA compound binding, while loss of m6A methylation was enriched in genes involved in catalytic activity, ion binding, coenzyme binding, and cofactor binding (Figures 4A,B). These results suggest that m6A has complicated roles in CI-AKI, with primary roles in metabolism, various pathways related to cell death, and oxidation.

**FIGURE 5:**
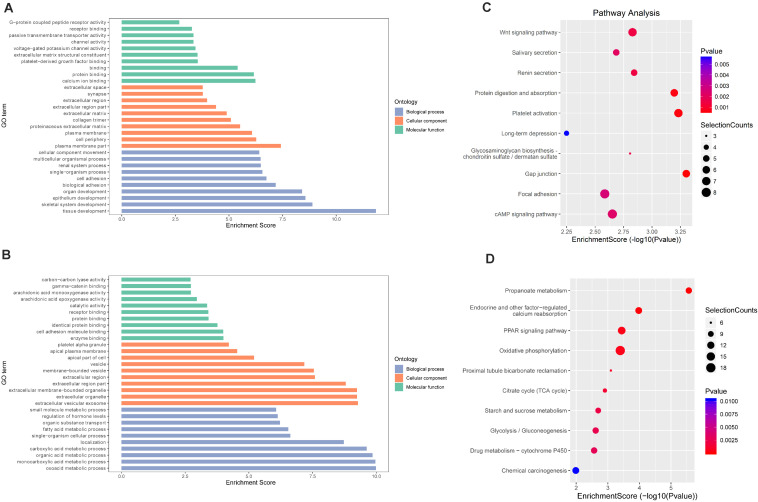
Gene ontology and Kyoto Encyclopedia of Genes and Genomes analyses of coding genes containing DMMSs in TvI. **(A)** Major gene ontology terms were enriched for the genes containing up-regulated m6A sites in TvI. **(B)** Major gene ontology terms were enriched for the genes containing down-regulated m6A sites in TvI. **(C)** Major enriched pathways for the genes containing up-regulated m6A sites in TvI. **(D)** Major enriched pathways for the genes containing down-regulated m6A sites in TvI.

In TvI (Figure 5), for the BP category, genes with increased methylation of m6A sites were significantly (*p* < 0.05) enriched in some development-associated processes, such as tissue, skeletal system, epithelium, and organ development (Figure 5A), while those with decreased methylation were highly enriched in acid metabolism processes, such as the oxoacid, organic acid, and carboxylic acid metabolic processes (Figure 5B). Regarding pathways, the former were significantly involved in gap junctions, protein digestion and absorption, and the Wnt pathway, while the latter were involved in metabolic processes, such as the PPAR signaling pathway (Figures 5C,D). For the CC category, genes containing DMMSs induced by cisplatin-induced AKI were mainly enriched in plasma membrane, cell periphery extracellular vesicular exosome, and extracellular organelles. For the MF category, increased methylation of m6A was notably enriched in calcium ion binding, protein binding, and channel activity, while decreased methylation of m6A was enriched in enzyme binding, cell adhesion molecule binding, and identical protein binding (Figures 5A,B). These results suggest that m6A methylation is involved in berberine alleviating CI-AKI in mouse kidneys.

**FIGURE 6:**
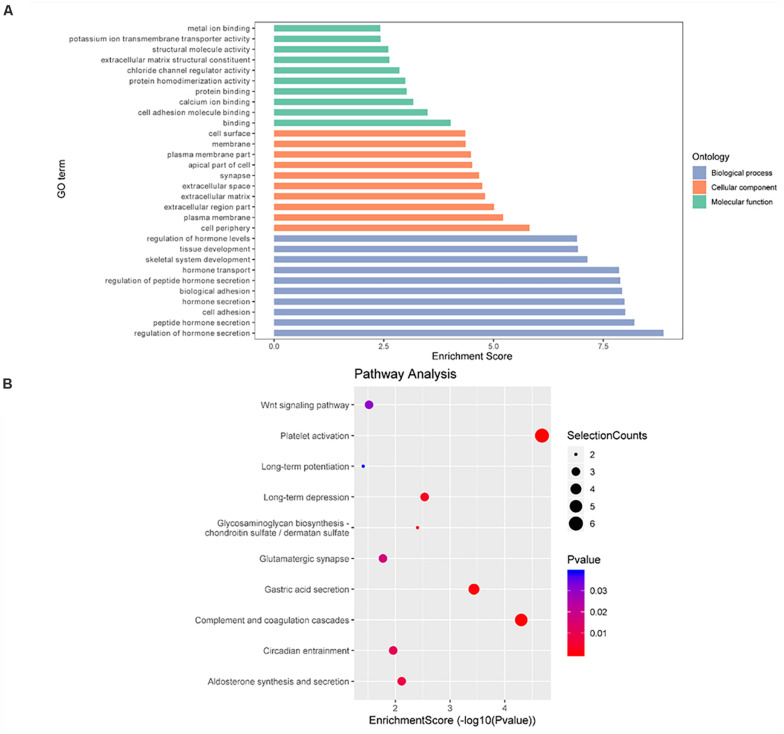
Gene ontology and Kyoto Encyclopedia of Genes and Genomes analyses of coding genes containing DMMSs. **(A)** Major gene ontology terms were enriched for the genes containing m6A sites with reverse methylation trend between IvC and TvI. **(B)** Major enriched pathways for the genes containing m6A sites with reverse trend between IvC and TvI.

Finally, GO enrichment and KEGG pathway analyses were conducted on genes containing m6A sites showing opposite methylation trends in IvC and TvI (Figure 6). For the BP category, genes with DMMSs were significantly (*p* < 0.05) enriched in hormone secretion processes, such as regulation of hormone secretion and peptide hormone secretion (Figure 6A). Pathway analyses demonstrated that genes with DMMSs were involved in platelet activation, complement and coagulation cascades, and gastric acid secretion (Figure 6B). For the CC category, genes containing DMMSs were mainly enriched in the cell periphery, plasma membrane, and extracellular matrix. For the MF category, genes were enriched in binding, cell adhesion molecule binding, and calcium ion binding (Figure 6A). These results suggest that berberine might resist the nephrotoxicity of cisplatin through different pathways.

### Conjoint Analyses of m6A Modification and Gene Regulation

**FIGURE 7:**
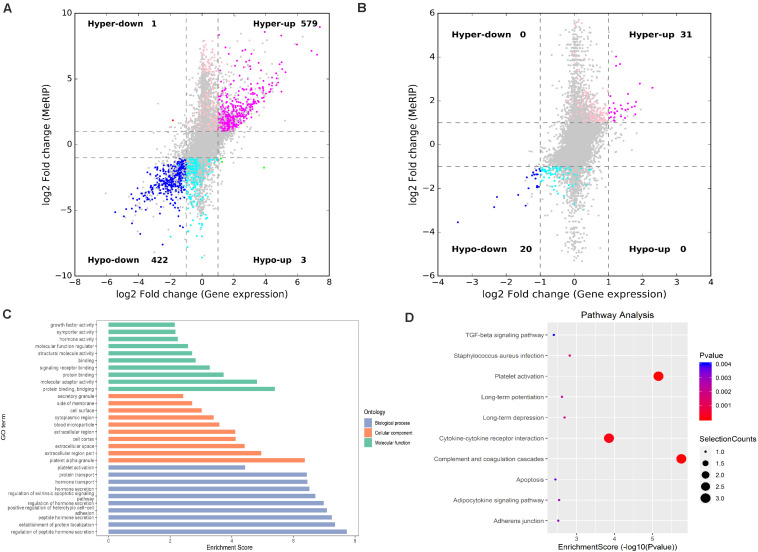
Conjoint analysis of differentially methylated genes and differentially expressed genes. **(A)** Four-quadrant graph exhibiting the DEGs containing differentially methylated m6A peaks in IvC. **(B)** Four-quadrant graph exhibiting the DEGs containing differentially methylated m6A peaks in TvI. **(C)** Major gene ontology terms were significantly enriched for the genes containing m6A sites with reverse methylation and expression trends between IvC and TvI. **(D)** Major enriched pathways for the genes containing m6A sites with reverse methylation and expression trends between IvC and TvI.

RNA-seq was used to detect DEGs among the groups. In IvC, 4,469 genes were differentially expressed (fold change ≥ 2 and *p* < 0.05), including 1,655 downregulated genes and 2,814 upregulated genes (Figure 7). Conjoint analyses of DMGs and DEGs resulted in four groups of genes: 576 hypermethylated and upregulated genes, 422 hypomethylated and downregulated genes, 1 hypermethylated but downregulated gene, and 3 hypomethylated but upregulated genes (Figure 7A). In TvI, 350 genes were differentially expressed, including 84 downregulated genes and 266 upregulated genes. Conjoint analyses revealed 31 hypermethylated and upregulated genes, 20 hypermethylated but downregulated genes, and no hypomethylated upregulated or hypomethylated downregulated genes (Figure 7B).

**FIGURE 8:**
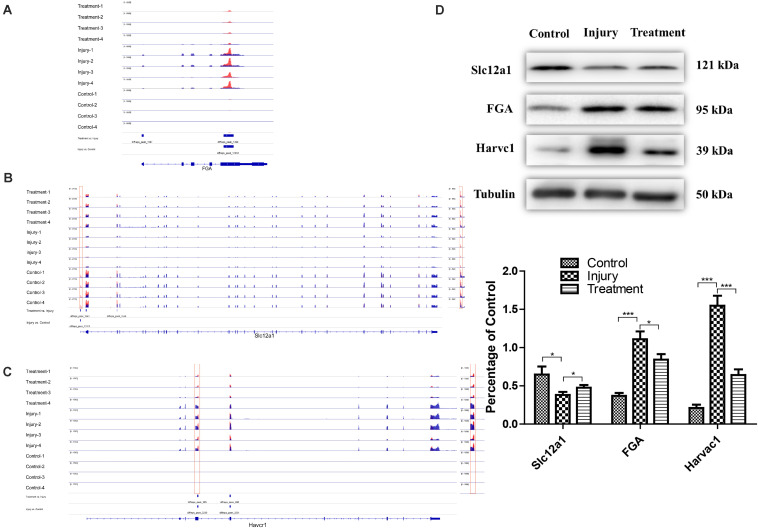
Representative genes with opposite m6A methylation and expression trends between IvC and TvI. **(A)** Visualization of m6A-modified genes FGA in the control group, injury group, and treatment group. **(B)** Visualization of m6A modified genes Slc12a1 in the control group, injury group, and treatment group. **(C)** Visualization of m6A-modified genes Havcr1 in the control group, injury group, and treatment group. **(D)** Western blot images of FGA, Slc12a1, and Havcr1 in control group, injury group, and Havcr1 group. Error bars represent the standard deviation. *p < 0.05, ***p < 0.001, Student’s *t*-test.

To further analyze the role of m6A in alleviating the action of berberine, genes with opposite methylation and expression trends in IvC and TvI were selected (Table 3). Overall, 9 were hypermethylated and upregulated in IvC but hypomethylated and downregulated in TvI, and 12 were hypomethylated and downregulated in IvC but hypermethylated and upregulated in TvI. GO and pathway analyses were performed to uncover the biological processes associated with these genes (Figure 7C). These genes were found to be highly enriched in complement and coagulation cascades, platelet activation, and cytokine–cytokine receptor interaction (Figure 7D). For instance, FGA/FGB/FGG genes encoding fibrinogen (Martini et al., 2019; Vilar et al., 2020) were hypermethylated and upregulated in IvC but were hypomethylated and downregulated in TvI (Figure 8A). Slc12a1, the key gene that mediates the electroneutral movement of Na(+) and K(+) across cell membranes (Schiessl et al., 2013; Xue et al., 2014; Hao et al., 2018; Kawaguchi et al., 2018), was hypomethylated and downregulated in IvC but hypermethylated and upregulated in TvI (Figure 8B). Havcr1, also known as Kim-1, is a well-known biomarker for kidney injury (Lippi et al., 2018; Seibert et al., 2018; Song et al., 2019; Zdziechowska et al., 2020). Cisplatin induced hypermethylation and upregulation of Havcr1, while berberine reversed these effects (Figure 8C).

**TABLE 3:** Genes with contrary methylation and expression trends between IvC and TvI.

Gene name	Gene ID	m6A regulation & gene regulation in IvC	m6A regulation & gene regulation in TvI	Chromosome	Peak start	Peak end	*P*-value in IvC	FDR in IvC	*P*-value in TvI	FDR in TvI
Akap12	83397	Up	Down	chr10	4353141	4353240	5.00E-05	0.00196624	5.00E-05	0.000154677
Amica1	270152	Up	Down	chr9	45107681	45108040	0.00335	0.052867	2.00E-04	0.000571614
Fga	14161	Up	Down	chr3	83031021	83031620	5.00E-05	0.00196624	5.00E-05	0.000154677
Fgb	110135	up	Down	chr3	83042246	83042340	5.00E-05	0.00196624	5.00E-05	0.000154677
Fgg	99571	Up	Down	chr3	83010063	83010180	5.00E-05	0.00196624	5.00E-05	0.000154677
Havcr1	171283	Up	Down	chr11	46756128	46756260	5.00E-05	0.00196624	5.00E-05	0.000154677
Inhbb	16324	Up	Down	chr1	119422161	119422248	5.00E-05	0.00196624	5.00E-05	0.000154677
Lep	16846	Up	Down	chr6	29073261	29073500	5.00E-05	0.00196624	5.00E-05	0.000154677
Mlph	171531	Up	Down	chr1	90950081	90950420	5.00E-05	0.00196624	5.00E-05	0.000154677
Egfl6	54156	Down	Up	chrX	166538413	166538734	5.00E-05	0.000154677	5.00E-05	0.00196624
Gbp4	17472	Down	Up	chr5	105118221	105118499	5.00E-05	0.000154677	0.00195	0.0357303
Gnaz	14687	Down	Up	chr10	75015621	75015980	2.00E-04	0.00635985	0.00045	0.00121049
Ppp1r1a	58200	Down	Up	chr15	103537841	103537992	5.00E-05	0.00196624	5.00E-05	0.000154677
Ranbp3l	223332	Down	Up	chr15	8967948	8968040	5.00E-05	0.00196624	5.00E-05	0.000154677
Slc12a1	20495	Down	Up	chr2	125152504	125152633	5.00E-05	0.00196624	5.00E-05	0.000154677
Slc16a7	20503	Down	Up	chr10	125230591	125230700	5.00E-05	0.00196624	5.00E-05	0.000154677
Snai2	20583	Down	Up	chr16	14708181	14708720	5.00E-05	0.00196624	5.00E-05	0.000154677
Tfap2b	21419	Down	Up	chr1	19212053	19212140	5.00E-05	0.00196624	5.00E-05	0.000154677
Tmem207	100043057	Down	Up	chr16	26504161	26504620	5.00E-05	0.00196624	5.00E-05	0.000154677
Tnfsf10	22035	Down	Up	chr3	27342154	27342427	5.00E-05	0.00196624	5.00E-05	0.000154677
Tril	66873	Down	Up	chr6	53818361	53819000	5.00E-05	0.00196624	5.00E-05	0.000154677

### Western Blotting Analyses of Protein Expression in FGA, Slc12a1, and Havcr1

Western blotting was conducted to detect the protein levels of FGA, Slc12a1, and Havcr1 in each group. Cisplatin induced an increase in Slc12a1 protein levels and a decrease in FGA and Havcr1 protein levels. However, berberine pretreatment reversed these effects (Figure 8D).

## Discussion

m6A methylation is considered a reversible dynamic modification in many species. Previous studies have shown that m6A modification can regulate cellular responses to stimuli by affecting mRNA transcription, splicing, localization, translation, stability, and posttranscriptional regulation of gene expression at the RNA level (Zhou et al., 2015; Fry et al., 2017). Evidence (Anders et al., 2018; Wang Y. et al., 2019; Zhou P. et al., 2019; Li et al., 2020; Liu et al., 2020; Xu et al., 2020) also suggests a strong relationship between m6A modification and kidney disease, thereby revealing an important biological role for m6A in the regulation of kidney injury.

In this study, a reliable cisplatin-induced AKI model was established in mice, and the model was tested by analyzing Scr, BUN, and kidney section images. The kidney injury resulted in dynamic m6A modifications and gene expression in the kidneys. Differentially methylated mRNAs were found to be involved in many biological pathways. m6A methylation was enriched in the CDS in all groups. In IvC, GO, and KEGG analyses of coding genes harboring DMMSs demonstrated that genes with increased methylation were primarily enriched in the pathways related to metabolic processes and cell death process, such as macromolecule metabolic processes, cellular metabolic processes, the TNF signaling pathway, and the MAPK signaling pathway, while decreases in methylation were mainly enriched in metabolic processes, oxidation, and transport, indicating that cisplatin induced complicated variation in m6A methylation. Several pathways have been thoroughly studied as factors affecting CI-AKI. However, we found that variation in metabolic processes constituted the foundation of the biological and pathological changes in CI-AKI.

Different m6A methylation sites were also detected in TvI. GO, and KEGG analyses of coding genes with DMMSs revealed that the genes with increases in methylation were primarily enriched in pathways associated with tissue, the skeletal system, and epithelium development, such as gap junctions and the Wnt signaling pathway. Genes with decreases in methylation were mainly enriched in the oxoacid metabolic process and organic metabolic process. These results provide evidence of a link between m6A and berberine-mediated regulation of kidney injury, showing that berberine attenuated CI-AKI by increasing the methylation of genes associated with tissue, the skeletal system, and epithelium development. These results should prove useful for directing future research into the underlying medical value of berberine in CI-AKI.

Furthermore, 94 DMMSs showed opposite methylation trends in IvC and TvI. The genes with those DMMSs were primarily enriched in pathways associated with hormone secretion and complement and coagulation cascades, which implies that berberine probably relieves CI-AKI by directly reversing some changes in m6A methylation.

Finally, conjoint analyses of m6A modifications and gene regulation provided a broad picture of CI-AKI and the impacts of berberine pretreatment. In total, 21 genes were found to show opposite m6A methylation and expression trends in IvC and TvI. Among these candidate genes, several drew our attention. For instance, FGA/FGB/FGG are the core genes encoding fibrinogen. Cisplatin may cause endothelial injury and inflammation, which can activate coagulation cascades (Ozkok and Edelstein, 2014). Thus, FGA/FGB/FGG were hypermethylated and upregulated in CI-AKI. However, berberine pretreatment significantly alleviated these trends. This result is consistent with one previous study (Riccioni et al., 2018). Slc12a1, also known as NKCC2, is the molecular target of loop diuretics, as it is expressed on the apical membrane of the thick ascending limb of Henle epithelial cells (Hao et al., 2018). It mediates the electroneutral movement of Na^(+)^ and K^(+)^, which is tightly coupled to the movement of Cl^(–)^ across cell membranes (Schiessl et al., 2013; Xue et al., 2014; Kawaguchi et al., 2018). In our study, Slc12a1 was hypomethylated and downregulated by cisplatin. Berberine pretreatment maintained transmembrane ion exchange by hypermethylating and upregulating Slc12a1. Havcr1, also known as KIM-1, is a promising biomarker for predicting kidney injury (Lippi et al., 2018; Seibert et al., 2018; Song et al., 2019; Zdziechowska et al., 2020). It may reduce acute injury by mediating phagocytosis (Yang et al., 2015). In our study, it was hypermethylated and upregulated in CI-AKI. However, berberine reversed these trends. Consistently, the protein expression levels of FGA, Slc12a1, and KIM-1 were similar to the m6A methylation and gene expression levels. These results suggest that berberine has a protective effect in CI-AKI through different pathways.

## Conclusion

We characterized the differential m6A methylome in normal, CI-AKI, and berberine-pretreated CI-AKI in mice. Our results suggest a strong association between m6A methylation and the regulation of CI-AKI. In addition, the candidate genes identified reveal the possible pathways by which berberine alleviates kidney injury. Altogether, our results provide a fundamental contribution to research into the mechanisms of and novel therapies for CI-AKI.

## Data Availability Statement

The datasets generated for this study can be found in (US National Center for Biotechnology Information Gene Expression Omnibus). The accession ID is GSE157261 (https://www.ncbi.nlm.nih.gov/geo/query/acc.cgi?acc=GSE157261).

## Ethics Statement

The animal study was reviewed and approved by The Institutional Animal Care and Use Committee of Shanghai Jiaotong University affiliated Renji Hospital.

## Author Contributions

ZN and XC contributed to design the research. JS, WW, and XS conducted the experiments. JS and WW analyzed and interpreted the data, and drafted the manuscript. JW and SL helped to polish the manuscript. All authors read and approved the final manuscript.

## Conflict of Interest

The authors declare that the research was conducted in the absence of any commercial or financial relationships that could be construed as a potential conflict of interest.

## Footnotes

1 www.geneontology.org

2 www.genome.jp/kegg

## Supplementary Material

Supplementary Figure 1 Venn diagram showing the overlap of m6A peaks within mRNAs in three groups.

Supplementary Figure 2 Chromosomal distribution of all DMM sites. **(A)** Chromosomal distribution of all DMM sites in IvC. **(B)** Chromosomal distribution of all DMM sites in TvI.

Supplementary Figure 3 Chromosomal distribution of DMM sites presenting contrary methylation trend between IvC and TvI.

## Abbreviations

m6A: N6-methyladenosine
CI-AKI: cisplatin-induced acute kidney injury
IvC: injury group vs. control group
TvI: treatment group vs. injury group
YTH: YT521-B homology
DEGs: differentially expression genes, negative control; i.p. injected intraperitoneally
Scr: serum creatinine
BUN: blood urea nitrogen
GO: Gene ontology
KEGG: Kyoto Encyclopedia of Genes and Genomes
CDS: coding sequence
MACS: Model-based Analysis of ChIP-Seq
DMMSs: differentially methylated m6A sites.
H-U: hyper-methylated and upregulated, 422 hypo-methylated and downregulated (hypo-down) genes, one hyper-methylated but down-regulated (hyper-down) genes and 3 hypo-methylated but up-regulated (hypo-up).
