# Supplementary figures and images for: Integrated Analysis of m6A Methylome in Cisplatin-Induced Acute Kidney Injury and Berberine Alleviation in Mouse
Source: Front Genet. 2020 Nov 20;11:584460. doi: 10.3389/fgene.2020.584460 (PMC7718005; doi:10.3389/fgene.2020.584460)

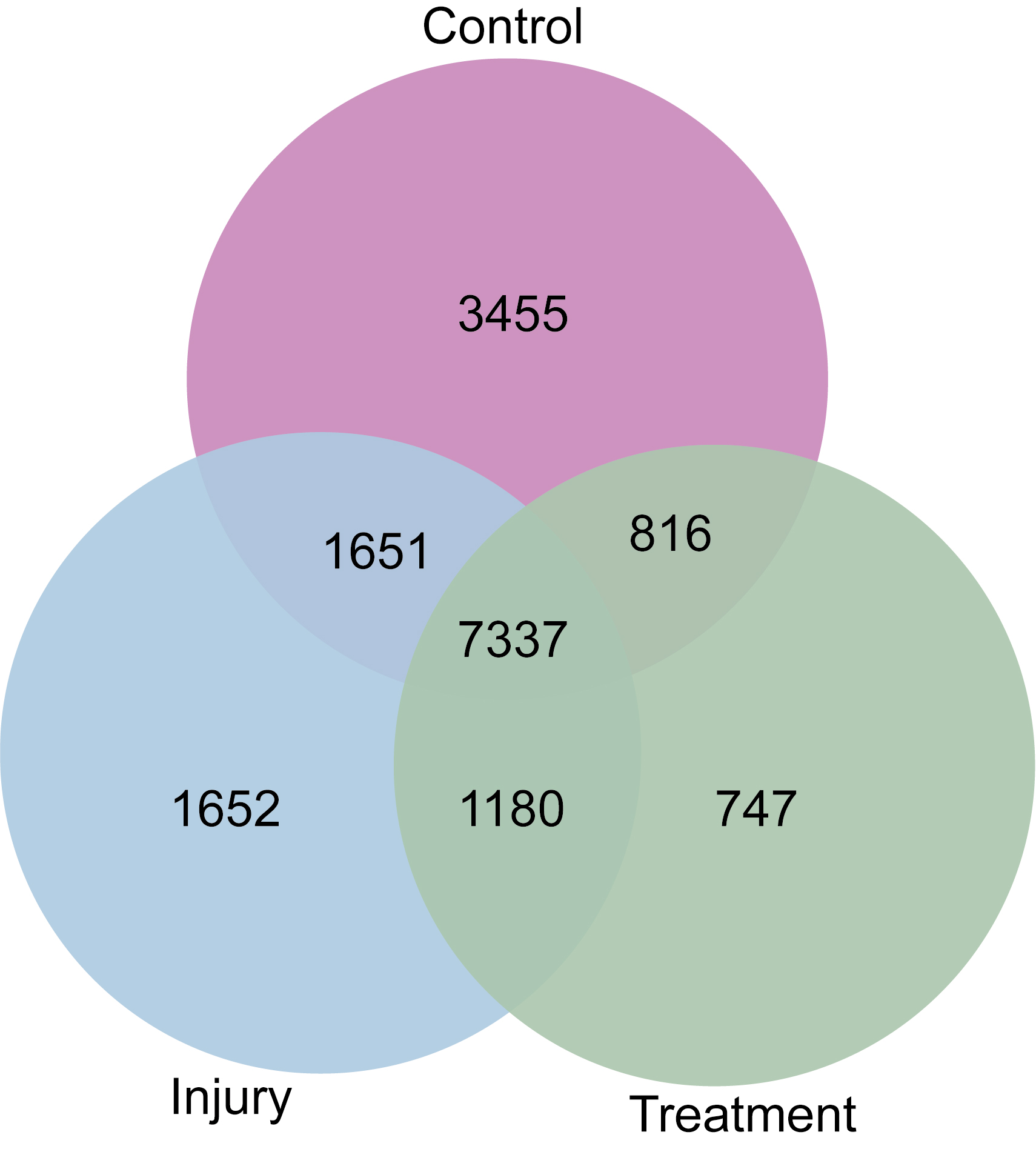

Supplement: Supplementary Figure 1 — Venn diagram showing the overlap of m6A peaks within mRNAs in three groups. [file Image_1.JPEG]

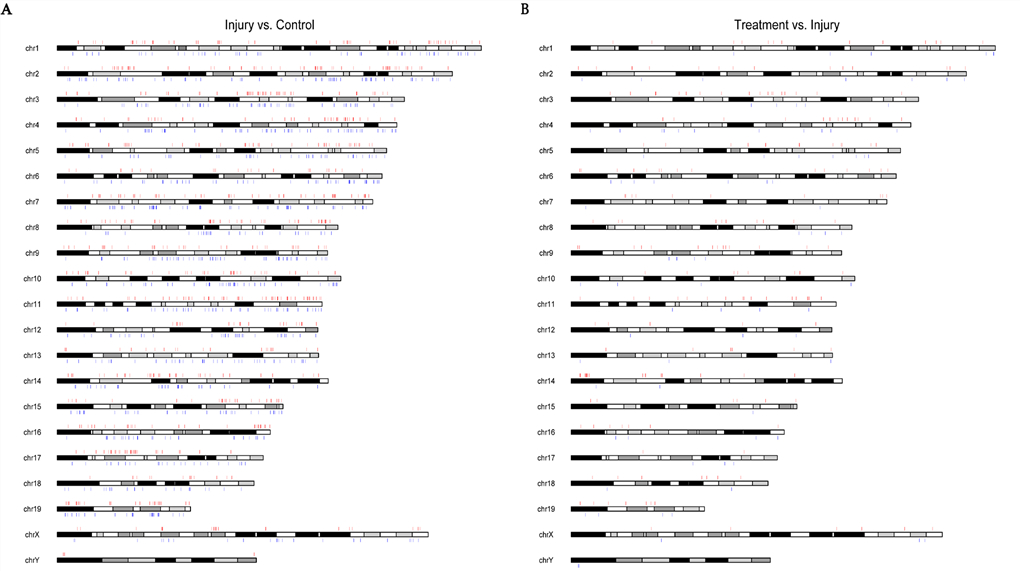

Supplement: Supplementary Figure 2 — Chromosomal distribution of all DMM sites. (A) Chromosomal distribution of all DMM sites in IvC. (B) Chromosomal distribution of all DMM sites in TvI. [file Image_2.JPEG]

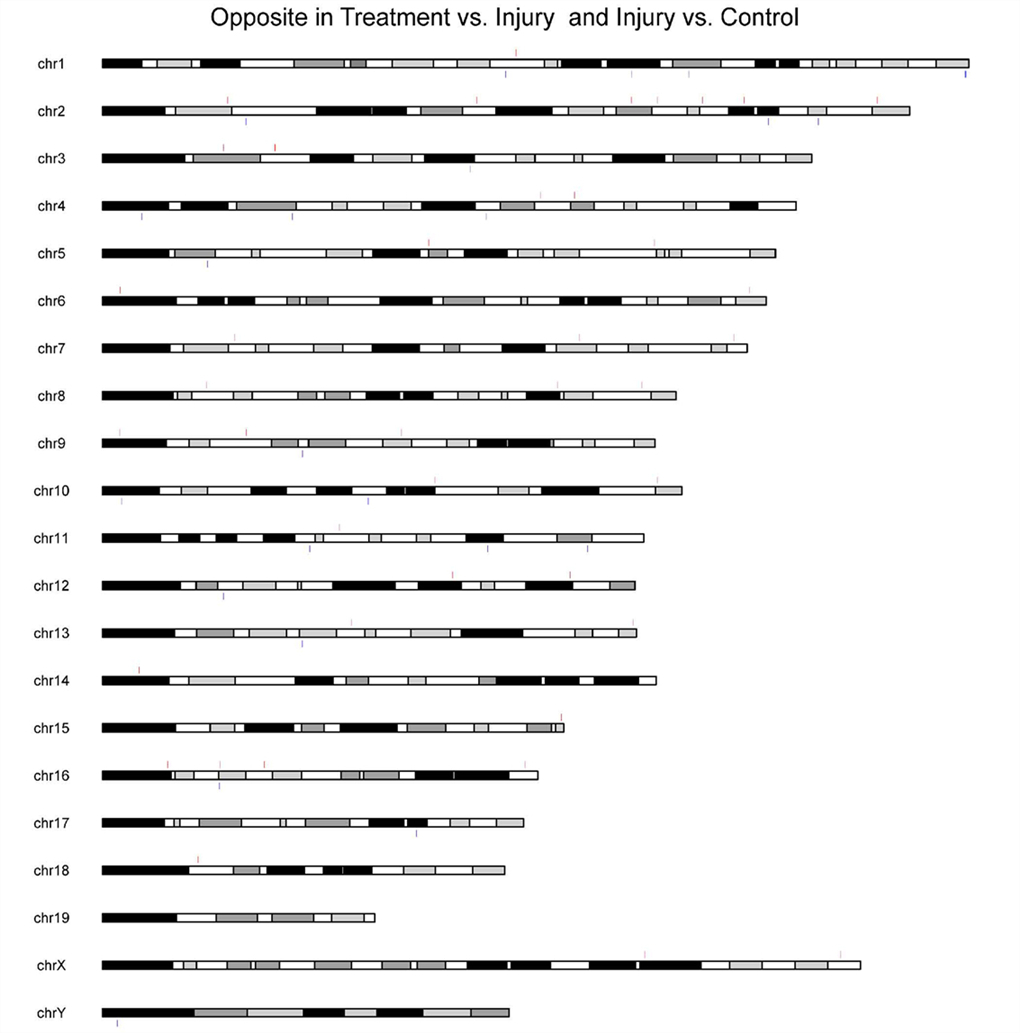

Supplement: Supplementary Figure 3 — Chromosomal distribution of DMM sites presenting contrary methylation trend between IvC and TvI. [file Image_3.JPEG]
